# Epididymal proteins Binder of SPerm Homologs 1 and 2 (BSPH1/2) are dispensable for male fertility and sperm motility in mice

**DOI:** 10.1038/s41598-020-66017-6

**Published:** 2020-06-02

**Authors:** Marzieh Eskandari-Shahraki, Bruno Prud’homme, Francis Bergeron, Puttaswamy Manjunath

**Affiliations:** 1 Maisonneuve-Rosemont Hospital Research Centre, Montreal, Quebec H1T 2M4 Canada; 2 Department of Pharmacology and Physiology, Faculty of Medicine, University of Montreal, Montreal, Quebec H3C 3J7 Canada; 3 Centre de recherche du CHU de Québec-Université Laval, 2705 Laurier Boulevard, Québec City, Québec G1V 4G2 Canada

**Keywords:** Protein translocation, Experimental models of disease

## Abstract

The binder of sperm family of proteins has been reported to be indispensable for sperm maturation and capacitation. However, their physiological functions in fertility have only been studied *in vitro*. CRISPR/Cas9 genome editing was utilized to generate double knockout (DKO) mice by simultaneously targeting the two murine binder of sperm genes, *Bsph1* and *Bsph2*. To confirm that the homologous genes and proteins were completely eliminated in the DKO mice, different methods such as reverse transcription polymerase chain reaction, digital droplet-polymerase chain reaction and liquid chromatography tandem mass spectrometry were applied. *Bsph1*/2 DKO male mice were bred by intercrossing. Compared to wild type counterparts, male *Bsph1/2* null mice, lacking BSPH1/2 proteins, were fertile with no differences in sperm motility and sperm count. However, the weights of male pups were significantly increased in *Bsph1/2* double knockout mice in a time dependent manner spanning days 6 and 21, as well as 6 weeks of age. No change was detected in the weights of female pups during the same period. Taken together, these data indicate that BSPH1/2 proteins are dispensable for male fertility in mice but may influence growth.

## Introduction

Mammalian fertilization is a multi-step process that arises from complex and regulated cellular processes, and relies on highly specialized gametes; defects in which can lead to infertility^1^. Despite numerous studies attempting to shed light on the molecular causes leading to human infertility, ambiguity remains. Several studies indicate that male and female factors contribute equally to cases of human infertility^2,3^. Thus, it is important to assess the causes of infertility in both sexes in order to apply the most effective fertility treatment^4^. Male fertility is affected by numerous factors of environmental, behavioral and genetic origin. However, in comparison to female infertility, male infertility has been investigated less thoroughly and its causes are less accurately documented^4,5^.

In mammals, sperm cells are produced in the testis and subsequently, immotile sperm enter the epididymis, progressively gaining their motility and ability to fertilize the oocyte while passing through the lumen of the different sections of this organ [reviewed in^6^]. Exposure to secreted proteins in the epididymal microenvironment results in morphological and biochemical alterations in sperm. The enormous diversity of proteins expressed in the epididymis provides the proper milieu for sperm maturation and the acquisition of fertilizing ability^7^. In order to study the role of epididymal proteins in male fertility, numerous genetically-engineered mouse models have been generated and analyzed [reviewed in^6^].

The Binder of SPerm (BSP) proteins constitute a conserved and ubiquitous family of mammalian sperm proteins, which are expressed in the seminal vesicles and/or epididymis depending on the species^8–10^. Two BSP homologs (BSPH1 and BSPH2) are found in mouse, while only one (BSPH1) has been identified in human^11^. The corresponding mouse homologous genes, *Bsph1* and *Bsph2*, are located on chromosome 7 and consist of five exons and four introns. The human *BSPH1* gene is located on chromosome 19 and encompasses six exons and five introns. The secondary structure of all proteins of the BSP superfamily contains two homologous and tandemly arranged domains, which are similar to the type-II domains present in fibronectin^10^. These fibronectin type-II domains (Fn2) specifically bind to choline phospholipids (PC) present in the sperm membrane, and have been suggested to promote sperm capacitation during sperm progression through the female reproductive tract^8^. Taking into consideration that murine and human BSP proteins share many biochemical and functional characteristics such as the organ in which they are expressed, their binding properties, and a similar structure^12^, studying BSP functions in mouse has the potential to provide invaluable information on the role of these proteins in human sperm function and fertility. Due to their co-localization on the same chromosome, generating a mouse model with the *Bsph1*/*Bsph2* double deletion via traditional knockout approaches presents important technical challenges. The development of new gene editing techniques has opened the door to powerful and attractive alternatives for investigating *in vivo* gene function. In the present study, we used CRISPR/Cas9 technology to generate a genetically-engineered mouse model in which the *Bsph1* and *Bsph2* genes were simultaneously deleted, and examined the phenotypes of the double knockout (DKO) mice. We then studied the effects of the absence of BSP proteins on sperm function and fertility. We observed that the absence of BSP family proteins in the *Bsph1/2* DKO did not cause male infertility or subfertility. However, the average weight of pups born from mating *Bsph1/2* DKO male mice with WT females was significantly increased compared to the WT. The present study is the first to investigate the endogenous functions of BSP proteins and their role in male fertility *in vivo*.

## Results

### Establishment and characterization of the *Bsph1/2* DKO mouse line

**Figure 1:**
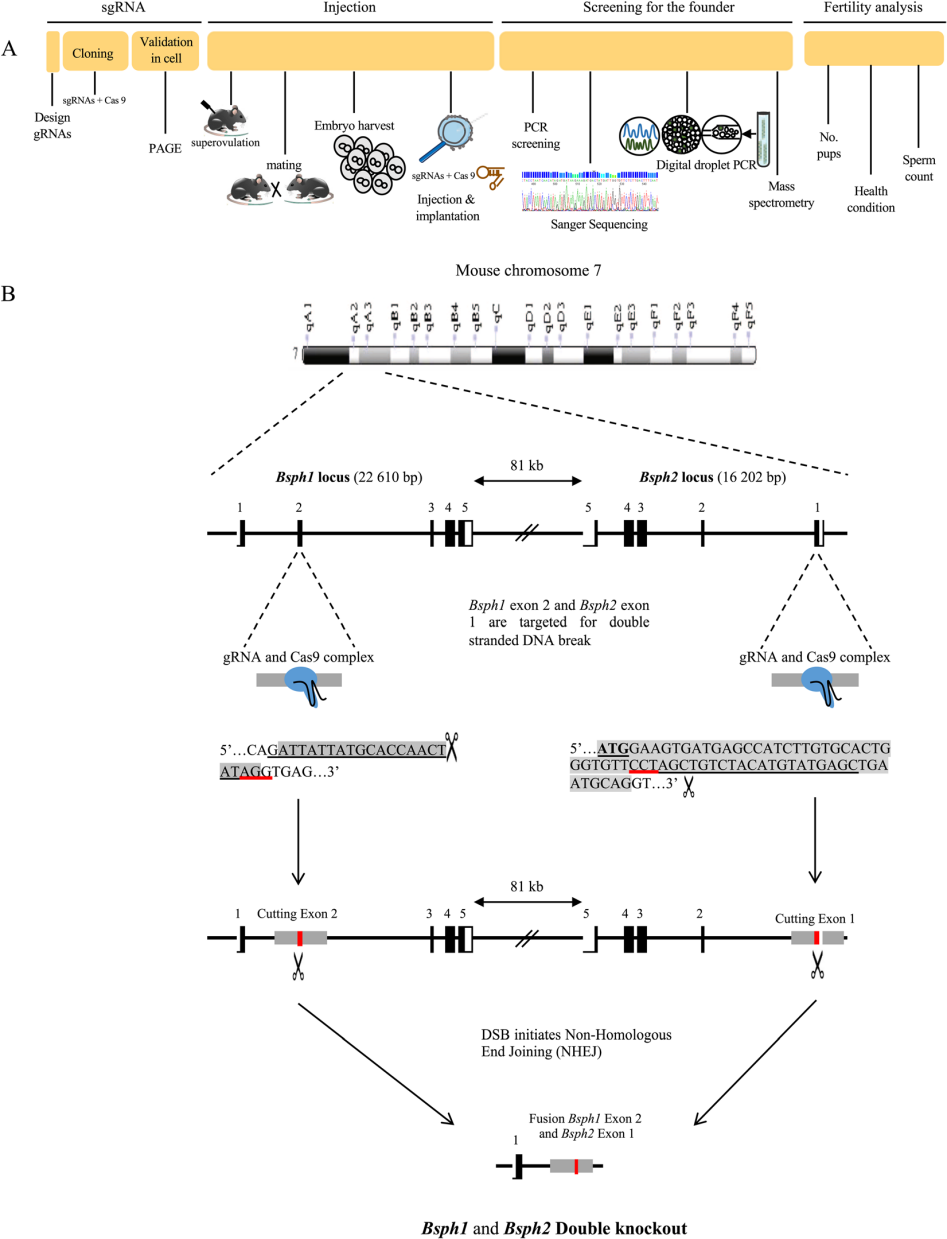
(**A**) Overall strategy for the design, generation, and analysis of *Bsph1/2* DKO mice. (**B**) Schematic representation of the *Bsph1* and *Bsph2* genomic locus in mouse and the CRISPR/Cas9 gene deletion strategy. For each gene, exons are shown (boxes) with untranslated (white) and coding (black) regions. A part of the DNA sequence of the targeted coding exons are shown (highlighted in grey), with the respective selected guide RNA sequences (underlined) associated with the PAM sequence (red underlined). Scissors indicate the theoretical positions where Cas9 creates the double stranded break (3-4 nucleotides upstream of the PAM). The non-homologous end joining (NHEJ) repair pathway creates random insertions or deletions (indels) at the cut site.

**Figure 2:**
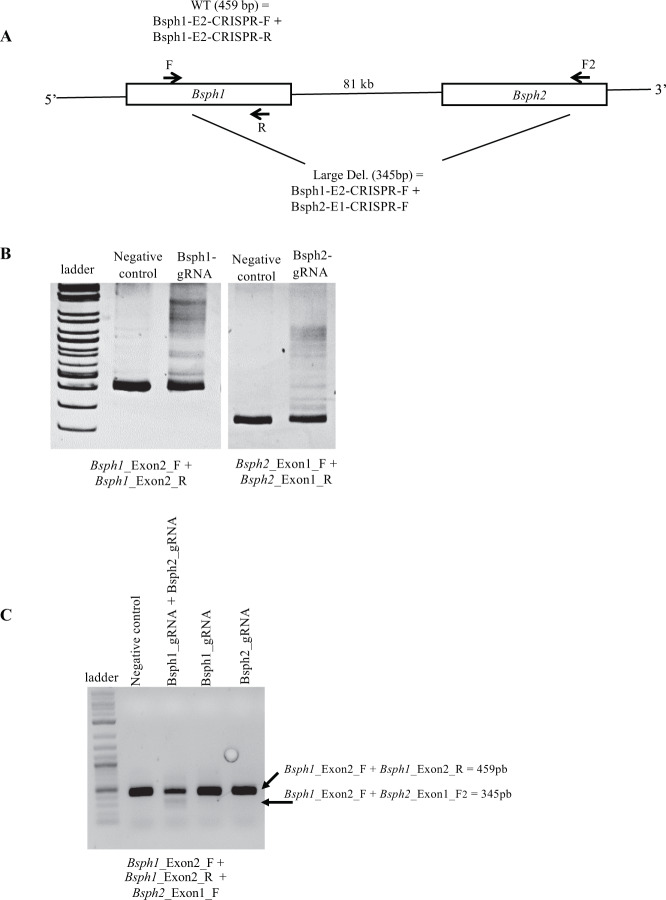
*In vitro* validation of *Bsph1* and *Bsph2* guide RNA efficiency in N2a cells. (**A**) PCR screening strategy to assess *Bsph1* and *Bsph2* guide RNA efficiency. A schematic representation of the *Bsph1* and *Bsph2* genomic locus is shown along with the positions of the different PCR primers that were used. (**B**) Indel mutations induced by CRISPR/Cas9 plasmids targeting the *Bsph1* or *Bsph2* genes are revealed by the presence of DNA heteroduplexes. (**C**) PCR amplification of genomic DNA in the presence or absence of plasmids containing guide RNAs to target the *Bsph1* and *Bsph2* genes. Simultaneous CRISPR/Cas9 targeting of the *Bsph1* and *Bsph2* genes results in a 115 kb deletion as a result of the fusion of *Bsph1* E2 and *Bsph2* E1. This is identified as a new 345-bp PCR product. Unsuccessful targeting, or targeting of *Bsph1* or *Bsph2* separately, will result in an expected 459-bp fragment common with a negative control plasmid targeting another genomic region. *Negative control*, treatment of genomic DNA with a plasmid targeting another genomic region.

The overall strategy used to generate and analyze *Bsph1*/*2* DKO mice is depicted in Fig. 1A. Exon 2 and exon 1 of the *Bsph1* and *Bsph2* loci, respectively were selected for targeting by CRISPR/Cas9 (Fig. 1B). The guide RNAs (gRNAs) were designed, synthesized and ligated into the pX330 plasmid to target the early sequence of exons 2 and 1 of the *Bsph1* and *Bsph2* genes, respectively. Before injecting the constructs into mouse embryos, the designed gRNAs were validated in neuroblast N2 cells to ensure that they could target efficiently and cut the desired regions of BSP genes. An overview of the *Bsph1* and *Bsph2* loci and PCR strategy used to assess guide efficiency is shown in Fig. 2A. Efficiency of the individual guides was first assessed by examining heteroduplex DNA formation in Neuro-2a (N2a) cells (Fig. 2B). Simultaneous targeting of both the *Bsph1* and *Bsph2* genes was then identified by PCR amplification of N2a genomic DNA transfected with plasmids containing both guide RNAs. Successful targeting of both genes generated a large 115-kb DNA deletion that was visualized as a new 345-bp fragment that resulted from the fusion of *Bsph1* E2 and *Bsph2* E1 (Fig. 2C). After validation, gRNAs along with Cas9 were microinjected into mouse two-cell embryos and transferred into pseudo-pregnant females. Microinjection of plasmid containing the gRNAs and Cas9 to target the *Bsph1* and *Bsph2* genes resulted in 31 newborn pups (12 males and 19 females).

**Figure 3:**
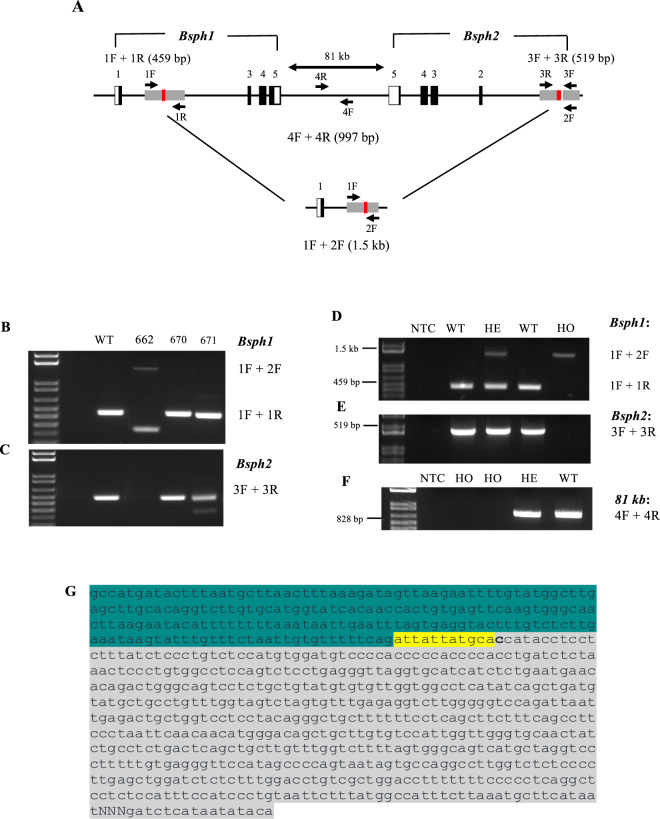
Characterization of the *Bsph1/2* DKO mouse line. Genomic DNA from tail snips of wild type (WT), double heterozygous (HE) and double homozygous (HO) mice was amplified by PCR using primers for *Bsph1*, *Bsph2* and the 81-kb linker between the two genes, as indicated. (**A**) Schematic representation of the *Bsph1/2* gene loci with overlaying primers (arrows) used for PCR amplification of the *Bsph1* and *Bsph2* genes. (**B,C**) PCR genotyping on genomic DNA from founder mice. Founder 662, was simultaneously targeted by CRISPR/Cas9 for both *Bsph1* and *Bsph2* genes. (**D**) Amplification using *Bsph1* primers resulted in bands of 1.5 kb and 459 bp for the mutated and WT genes, respectively. (**E**) Amplification using *Bsph2* primers generates a 519 bp fragment for WT and no product for *Bsph1/2 DKO*. (**F**) Amplification using 81 kb primers generates a 828-bp fragment for WT and no product for *Bsph1/2* DKO. (**G**) DNA sequence of the *Bsph1/2* DKO mouse. Sanger sequencing indicated that only part of *Bsph2* exon 1 (grey) and *Bsph1* exon 2 (green) remains in DKO mice (the rest of *Bsph1* and *Bsph2* as well as the 81 kb linker between have been removed). The green, yellow and grey represent the remaining parts of *Bsph1*, targeting site of gRNA, and *Bsph2*, respectively.

To investigate gene modifications at the target loci, primers were designed (Table 1) to amplify the respective targeted sites for both *Bsph1* and *Bsph2* (Fig. 3A). In order to consider all the probabilities for gene modification, a combination of three primers (listed in Table 1) were used to screen for *Bsph1*. For the *Bsph1* gene, pairs of forward and reverse primers (1 F and 1 R) were designed to specifically amplify the gene region targeted by CRISPR. Successful amplification using this primer pair would indicate that the allele is WT. However, the third primer (2 F) of *Bsph2* assists to recognize if the sequence between two targeted sites has been removed by CRISPR/Cas9 (Fig. 3A). Therefore, the PCR amplicon sizes for WT and DKO alleles were 459 bp and 1.5 kb, respectively. To amplify the *Bsph2* gene, a pair of primers (3 F and 3 R; see Table 1) was used; the expected amplification products are a single 519 bp band for WT and no product for KO as the complementary sequence for the reverse primer is lost (Fig. 3A). Genotyping showed that 8 of 31 founders were targeted by CRISPR/Cas9 for both genes (*Bsph1* and *Bsph2*) (Fig. 3B,C). To identify a line containing the *Bsph1/2* double deletion on the same homologous chromosome, six founders (2 males and 4 females) were backcrossed with C57BL/6 wild-type (WT) animals to establish heterozygous *Bsph1/2* DKO mice. Heterozygous offspring were interbred, and PCR analysis (Fig. 3D–F) followed by Sanger sequencing (Fig. 3G) showed that the CRISPR/Cas9 editing resulted in the elimination of the entire sequence between exon 2 and exon 1 of the *Bsph1* and *Bsph2* genes.

**Table 1:** Oligonucleotides used in this study.

*Primers*	*Sequences (5*′-*3*′*)*	*Experimental use*
Bsph1-E2-CRISPR-F2^a^	AGGCCACTGGACTAGAGTCAT	PCR
Bsph1-E2-CRISPR-R2^a^	ACAGCAGGCACAAGACCATT	PCR
Bsph2-E1-CRISPR-F^b^	GGCAAGGTATGCTCCTGTGT	PCR
Bsph2-E1-CRISPR-F2^b^	GCAAAACCAAAACCTCCCCA	PCR
Bsph2-E1-CRISPR-F^C^	GGCAAGGTATGCTCCTGTGT	PCR
Bsph2-E1-CRISPR-R^C^	GACCAAGGCTCCGTCATAGG	PCR
R81s-F^D^	CCTGCAATGCAGATTCCAACATATTGCC	PCR
R81s-R^D^	GGTGAGAGCCACATGTATTTGTGGGTCC	PCR
RT-mBsph2-E1F	AGTAGCCATCTTGTGCACTGG	RT-PCR
RT-mBsph2-E4R	CCTCCTTGGTGCACTTCTTAATGA	RT-PCR
mHprt-F	TCCTCCTCAGACCGCTTTT	ddPCR
mHprt-R	CCTGGTTCATCATCGCTAATC	ddPCR
qPCR-Bsph1-F5	AGTAGAAATCTCTTGTTCTGGAGG	ddPCR
qPCR-Bsph1-R5	AGTAGAAATCTCTTGTTCTGGAGG	ddPCR
qPCR-Bsph2-F6	GGGTGTTCCTAGCTGTCTAC	ddPCR
qPCR-Bsph2-R6	GGAGGGAGATACAACTGTAGTGG	ddPCR

^a^Referred to as primers 1F and 1 R in Fig. 4A.

^b^Referred to as primers 2 F in Fig. 4A.

^C^Referred to as primers 3 F and 3 R in Fig. 4A.

^D^Referred to as primers 4 F and 4 R in Fig. 4A

**Figure 4:**
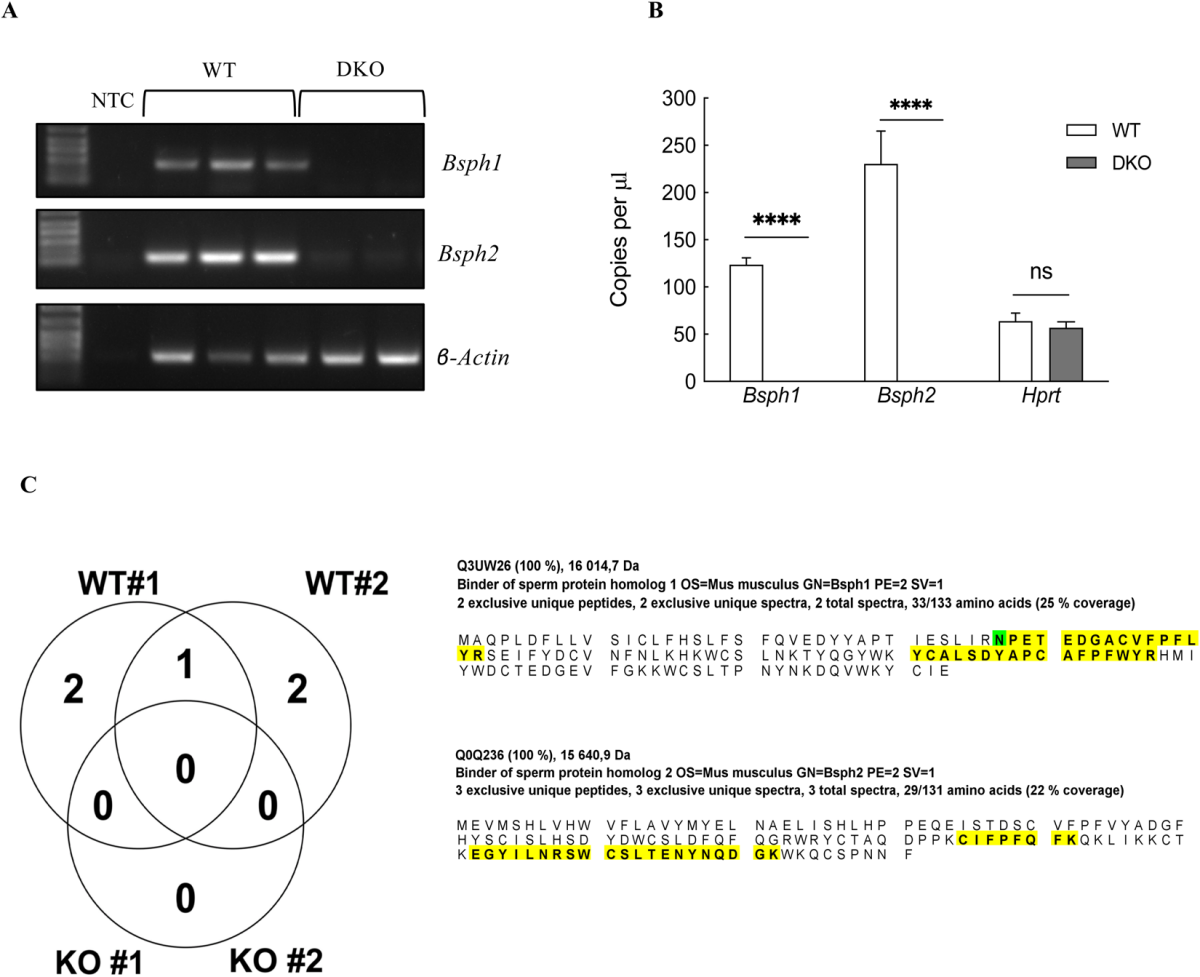
(**A**) RT-PCR analysis of epididymal RNA from WT and *Bsph1/2* DKO mice using primers for *Bsph1*, *Bsph2* and the 81-kb linker sequence. No template (NTC) was used as a control. (**B**) Expression of *Bsph1* and *Bsph2* genes in WT and *Bsph1/2* DKO epididymis as evaluated by ddPCR. *Bsph1* and *Bsph2* expression in DKO was compared with WT and the *Hprt* gene was used as a reference. Data are shown as the mean ± SEM (n = 3). Asterisks indicate a statistically significant difference. (***P < 0.001). (**C**) LC-MS/MS analysis of epididymal sperm protein extracts from WT and *Bsph1/2* DKO mice. Peptide sequences for BSPH1 and BSPH2 detected in WT by LC-MS/MS are shown in yellow. The amino acid in green (N) shown by Scaffold software highlights the modified amino acid (from the original sequence of the database, that is deamidation of Asparagine). No peptide sequences for BSP proteins were detected in protein extracts from DKO mice compared to WT as shown in the left panel.

Next, reverse transcription polymerase chain reaction (RT-PCR) was performed using cDNA synthesized from the epididymides of sexually mature male mice to explore the expression pattern of *Bsp* transcripts in the gene disrupted mice. As expected, *Bsph1* and *Bsph2* transcripts were not detected by RT-PCR in homozygous *Bsph1/2* DKO mice (Fig. 4A). Consistent with the RT-PCR results, digital droplet-polymerase chain reaction (ddPCR) analysis showed that *Bsph1* and *Bsph2* transcripts were also absent in homozygous *Bsph1/2* DKO mice (Fig. 4B). Finally, to prove that BSP proteins are also absent in *Bsph1/2* DKO mice, LC-MS/MS analysis was performed on WT and DKO epididymal protein extracts. The peptides obtained from LC-MS/MS analysis of WT epididymal protein extracts were compared to those obtained from *Bsph1/2* DKO extracts. As shown in Table 2 and Fig. 4C, neither BSPH1 nor BSPH2 peptides were detected by LC-MS/MS in epididymal extracts from the *Bsph1/2* DKO mice. We used a standard 1% false discovery rate on peptides and protein and found two BSPH1 peptides (Q3UW26) and three BSPH2 peptides (Q0Q236) only in epididymal extracts from WT mice. No BSP proteins were detected in epididymal protein samples of DKO mice by LC-MS/MS even if the level of confidence was decreased to 20%.

**Table 2:** Peptide sequences found for BSPH1 and BSPH2 in WT and *Bsph1/2* DKO mice.

Band No.	Protein ID’s	Mol. wt. (kDa)	Quantitative Value (Norm. total spectra)	Sequence Coverage (%)	Unique Peptide reads	Unique Spectrum matched	Peptide sequence matched
WT # 1	BSPH1	16	2	25	2	2	NPETEDGACVFPFLYR YCALSDYAPCAFPFWYR
WT # 1	BSPH2	15.6	1	6	1	1	CIFPFQFK
WT # 2	BSPH2	15.6	3	22	3	3	CIFPFQFK EGYILNR SWCSLTENYNQDGK
DKO # 1	—	—	0	0	0	0	—
DKO # 2	—	—	0	0	0	0	—

### *Bsph1/2* DKO male mice have normal sperm parameters

**Figure 5:**
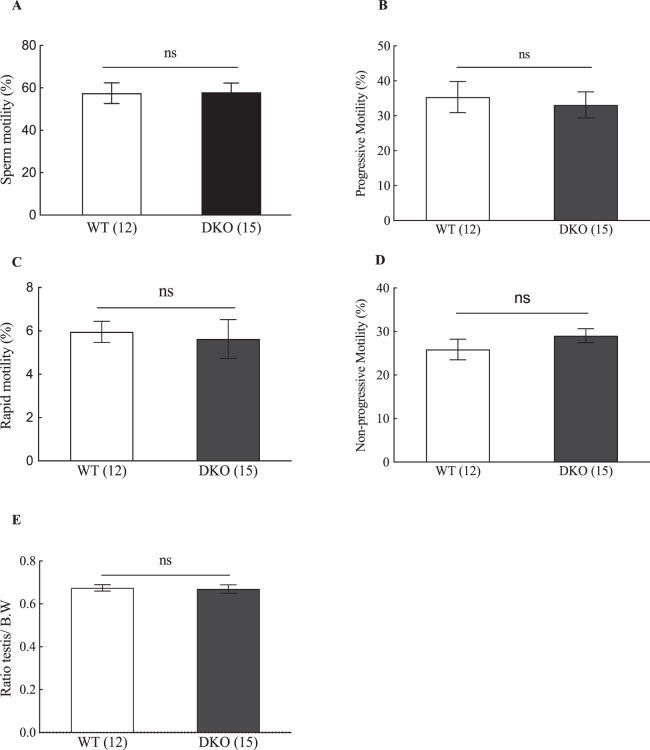
Analysis of *Bsph1/2* DKO and WT epididymal sperm function and fertility **(A)** Sperm motility, **(B)** Progressive motility, **(C)** Rapid motility, (**D)** Non-progressive motility, n = 7. (**E)** Ratio of testis to body weight (B.W.). Data are mean ± SEM; the number of males analyzed (n) is specified in brackets.

To determine whether the absence of BSP proteins had an effect on sperm functional parameters, sperm from six-week-old *Bsph1*/*2* DKO and WT mice were collected and analyzed by a Sperm Class Analyzer (SCA) system. No significant differences were observed between WT and *Bsph1/2* DKO mice with regards to sperm motility (Fig. 5A), percentage of progressive motility (Fig. 5B), rapid motility (Fig. 5C), and non-progressive motility (Fig. 5D). No obvious abnormalities were observed in the sperm morphology or sperm counts between *Bsph1/2* DKO and WT mice. The appearance and weights of the testes of WT and *Bsph1*/*2* DKO mice were also comparable (Fig. 5E).

### *Bsph1/2* DKO male mice have increased body weight but exhibit normal fertility

**Figure 6:**
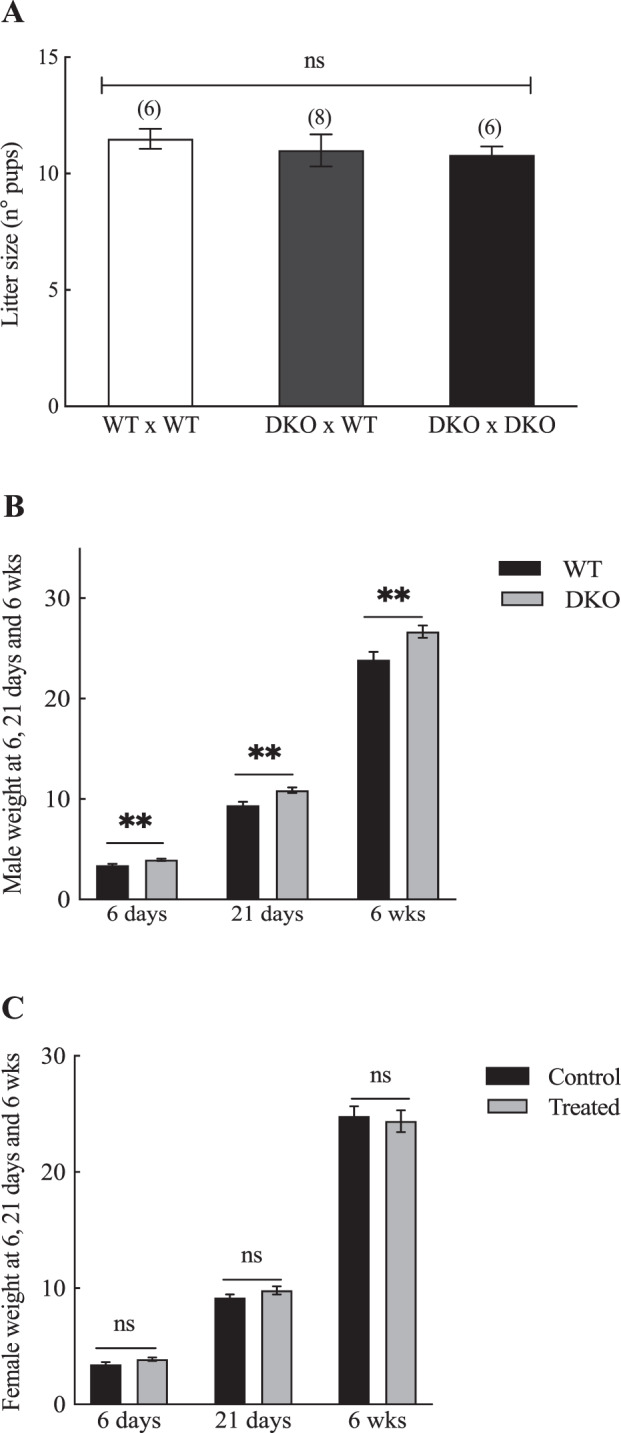
Comparison of the body weight of WT and DKO pups at 6 and 21 days, and 6 weeks. (**A**) WT or DKO adult males were bred with control females and the number of pups was analyzed. Data are mean ± SEM; the number of males analyzed (n) is specified in brackets. (**B**) Weights of male pups in a time dependent manner spanning days 6 and 21, as well as 6 weeks of age. (**C**) weight of female pups born during the same period of time. Data are presented as the mean ± SEM of eight independent experiments. Differences relative to WT (control) were analyzed by GraphPad Prism 7. (*P < 0.05, **P < 0.01, ***P < 0.001).

To investigate the effect of the absence of BSP proteins on male fertility, trio mating was performed by either mating one mutant male mouse with two WT female mice, or one WT male with two WT females for two weeks and the number of delivered pups was analyzed. Six-week-old male *Bsph1/2* DKO mice were fertile although the number of pups produced was slightly less when compared to the WT control group (Fig. 6A). The mating experiments resulted in the birth of a comparable number of pups from WT and *Bsph1/2* DKO male mice (Fig. 6A). To investigate whether the simultaneous absence of BSP proteins in both male and female was affecting animal fertility, DKO males were bred with DKO females. No further decrease in fertility was observed when DKO males were bred with DKO females (Fig. 6A). More matings were carried out using older *Bsph1/2* DKO male mice with WT females to determine whether the absence of BSP proteins could affect the fertility of aged male mice. However, no significant differences were observed in terms of fertility between the knockout and WT aged mice (data not shown). In addition, gestation time of WT females impregnated by *Bsph1/2* DKO males was comparable to that of WT females impregnated by WT males (data not shown). In our breeding analysis of *Bsph1/2* DKO mice, no obvious abnormalities in pup development were observed when compared to the WT group. Interestingly, despite no obvious effect on fertility, the body weight of male offspring at 6 days, 21 days (weaning) and 6 weeks of age was significantly increased when *Bsph1/2 DKO* males were bred with WT females compared to those sired from WT males (Fig. 6B). However, this was not seen with the female offspring sired from *Bsph1/2 DKO* males with WT females as no statistically significant differences were detected between the two groups (*P* > 0.05) (Fig. 6C).

## Discussion

Many molecules and proteins are suspected to play a role in fertility. Determining whether expression of specific genes is required for fertility could help to improve the diagnosis of infertility and select the best option for clinical treatment. Gene knockout (KO) is used to determine whether the absence of one or more genes leads to impacts *in vivo*^13^. In addition, mutation of genes expressed only in reproductive organs often does not affect animal viability, which makes these types of studies worthwhile to pursue. Many *in vitro* studies have characterized BSP proteins and shown that these proteins are present only in the male reproductive tract, suggesting that they could play an important role in sperm function in human and rodents^14^. Gene KO would thus be an effective strategy to determine whether the presence of BSP family proteins is essential for fertility *in vivo*^15^. In this study, we used CRISPR/Cas9 gene editing to target the two BSP genes in mouse, *Bsph1* and *Bsph2*, in order to investigate their role in male fertility. Despite their proposed role in sperm maturation and capacitation, we show that the BSP proteins are not required for fertility, at least in mice.

Previous data showed that BSP proteins bind over the midpiece of the flagellum, suggesting that family of proteins may play role in sperm motility^12^. However, when motility was examined, *Bsph1/2* DKO spermatozoa have normal motility parameters. Total sperm motility, percentage of progressive motility, rapid motility, and non-progressive motility were similar to WT sperm. These data suggested that depletion of BSP proteins did not affect the motility and *Bsph1/2* DKO knockout spermatozoa have normal motility.

In previous study, using the advanced technology of CRISPR/Cas9 we have targeted the *Bsph2* gene in mice^14^. Results showed that these mutant male mice lacking BSPH2 protein were fertile and had normal sperm parameters. However, lack of observing phenotype in single *Bsph2* KO and existence of the other member of BSP family, BSPH1, reinforced the existence of compensation from a homolog, which could be the reason why the single *Bsph2* KO was fertile^14^. However, increased weights were observed solely in *Bsph1/2* DKO male mice and no significant increased weights were observed in single *Bsph2* KO mice.

In terms of fertility, our past^14^ and current *in vivo* results indicate that BSP proteins, either individually or together, are not essential for sperm maturation and fertilizing ability in mice^14^. Interestingly, investigation of other fertilization related genes by gene disruption technology demonstrated that many of these genes, despite being shown to be essential *in vitro*, were surprisingly found to be dispensable for fertility *in vivo*^16^. These include the genes for *Acr* (acrosin)^17^, *β4galt1* (GalTase)^18^, *Adam1a/b* (Fertilin)^19^, *Crisp1*^20^ and many other fertilization related factors^16,21^. At present, only a handful of fertilization related factors such as IZUMO1^22^ and Adam2^23^ are known to be essential for fertilization.

A fertility study by Miyata *et al*. analysed the necessity of 54 conserved testis genes for fertility in mice. Results indicated that despite the evolutionary conservation of the genes, the absence of these genes had no significant impact on male fertility^24^. Hundreds of genes are expressed in the reproductive tract and the potency of gene manipulation techniques now gives us the ability to discover the function and importance of each gene. Despite the lack of an apparent phenotype in some KOs, it is essential to share the phenotypic data to avoid unnecessary redundant experiments and expenditures^24^. Furthermore, a deep systematic analysis of mouse sperm isolated from the cauda epididymis by Chauvin *et al*. led to the identification of 2850 proteins, which is the most comprehensive and complete proteome in cellular pathways encounter, and some of which might share functions with others to form an essential epididymal proteins set^25^. Therefore, the absence of one family of proteins such as BSPs might be rescued by other proteins working in parallel, thereby preventing the appearance of any phenotype^14,25^. Therefore, the redundancy of the genes involved in male reproduction may compensate for the absence of the mutated genes in the KO and DKO mice, thus preventing our observation of an infertility phenotype. Therefore, it remains a tedious challenge to discover the genes that are working in parallel to enable fertilization, since many share similar functions and can serve as a backup for one another.

A lack of a male reproductive phenotype in the *Bsph1/2* DKO mice was not unexpected. Data from a study by Fan *et al*.^26^, showed that BSP proteins in certain species such as mice and human are present in very low quantities, whereas they constitute around sixty percent of seminal plasma proteins in other species such as bovine. This suggests that BSP proteins may simply play different roles in different species^26,27^. Based on numerous KO studies, it seems that genetic redundancy also plays an important role in the reproductive system to minimize the effects on fertility caused by deficiencies and/or mutations in one or more genes. This could also be the reason why no obvious phenotype was observed in the majority of KO mice produced for other epididymal genes such as *Wdr63*^28^ and *Dnaic1*^29^. Another possible explanation is genetic redundancy. In this study, both the *Bsph1* and *Bsph2* genes were deleted and no subfertility or infertility phenotype was observed. This could be attributable to the existence of genes encoding proteins with similar functions to BSP proteins, but with unrelated sequences. Thus, it is possible that for male infertility to arise, mutations or dysregulation must be present in more than one family of genes. It would be fundamental to assess groups of genes in the infertile male to help pinpoint the causes of infertility.

Interestingly, the weight of heterozygous male pups born from WT females sired by *Bsph1/2* DKO males was significantly higher compared to those born from WT females sired by WT males. However, the weight of female pups was not different compared to the control group. This was an unexpected result that may suggest that the BSP genes and their encoded proteins could play a role in growth and weight regulation in males. At present, the explanation for this observation is unknown. However, this male bias is consistent with the fact that BSP proteins are specific to males^11^. We can perhaps speculate that BSP proteins might regulate androgen action in developing males. Previous studies showed that the 81 kb DNA segment between *Bsph1* and *Bsph2* in mice appears to include binding sites for the androgen receptor (AR). However, due to our deletion, the 81 kb linker between *Bsph1* and *Bsph2*, which is the site for androgen binding, has been removed and this has probably led to the removal of the androgen binding receptor(s) in *Bsph1/2* DKO mice. Indeed, alterations in androgen homeostasis, including that of testosterone and/or androstenedione, their secretion, transport and/or binding have been shown to cause profound changes in metabolism and predispose males to obesity^30,31^. Our results, therefore, form the basis for further studies to examine this potentially important and unexplored role for BSP proteins in males.

## Conclusion

Our findings demonstrate that epididymal BSP proteins are dispensable for male fertility and do not fulfill critical roles in *in vivo* fertilization in mice on their own. As revealed by recent gene knockout studies^32^, many fertilization factors have also been shown to be dispensable for fertility, likely due to the presence of redundant mechanisms. While we have found that BSP proteins are also non-essential for fertility in mice; however, they may potentially have a role in the regulation of body weight and growth in males.

## Materials and methods

### Animals

C57BL/6 mice were purchased from the Jackson Laboratory (Bar Harbor, ME, USA) and maintained in a temperature-controlled (22 ± 1 °C), light-controlled (a light cycle of 12 h light: 12 h dark), and pathogen-free environment. All mouse experimental work was carried out according to the guidelines of the Canadian Council of Animal Care (CCAC). Mouse protocols were approved by the Maisonneuve-Rosemont Hospital animal care committee (#2017-SE-020).

### Generation of *Bsph1*/*Bsph2* DKO mice

A CRISPR design web tool (www.crispor.tefor.net) was first used to identify suitable regions on the mouse genome to target exon 2 and exon 1 of the *Bsph1* and *Bsph2* genes, respectively (Fig. 2). Guide RNA (gRNA) sequences were selected to optimize their predicted low, overall off-target potential. Primers corresponding to the two chosen guide sequences were cloned into the pX330-U6-Chimeric_BBCBh-hSpCas9 (#42230) plasmid was kindly provided by Dr. Qinzhang Zhu. Primers for the *Bsph1_*gRNA were: forward, 5′-caccgATTATTATGCACCAACTAT-3′; reverse, 3′-cTAATAATACGTGGTTGATAcaaa-5′. Primers for the *Bsph2_*gRNA were: forward, 5′-caccgCTCATACATGTAGACAGCT-3′; reverse, 3′-cGAGTATGTACATCTGTCGAcaaa-5′. The SpCas9/chimeric gRNA constructs were first validated in the neuroblastoma N2a cells line to generate the desired deletion in genomic DNA. The validated guide constructs were then microinjected into fertilized B6C3F1/J mouse eggs using the Microinjection and Transgenesis service of the Institut de Recherches Cliniques de Montréal (IRCM). Genomic DNA was extracted from tail snips using the AccuStart II Mouse Genotyping Kit (Quanta Bioscience, Beverly, MA, USA) following the manufacturer’s protocol. PCR genotyping was established using the primer pairs indicated in Table 1 and the following conditions for both *Bsph1* and *Bsph2*: 94 °C for 3 min, followed by 30–35 cycles of 94 °C for 30 s, 62 °C and 72 °C for 30 s each. The guide RNAs along with pX330 plasmid were injected into the pronucleus of a zygote as described previously^14^.

### RT-PCR and ddPCR

RNA was extracted from epididymides of six-week-old male mice using TRIzol™ reagent (Invitrogen, Carlsbad, CA, USA) according to the manufacturer’s instructions. The Agilent RNA 6000 Nano Kit was used to assess the quality of freshly extracted RNA and RNA was stored at –80 °C. The iScript cDNA synthesis kit (Bio-Rad, Hercules, CA) was used to synthesize cDNA from RNA as described previously^14^. Prepared cDNA was subjected to digital droplet-PCR ddPCR using the primers shown in Table 1 to assess *Bsph1* and *Bsph2* gene expression in WT and *Bsph1*/*2* DKO mice. In this study, the ddPCR system included a droplet generator and reader from Bio-Rad, (QX200 Droplet Digital PCR, Bio-Rad, Hercules, California, USA), which fractionates samples into ∼20,000 droplets. The ddPCR method was performed according to the manufacturer’s instructions with some modifications as described below. Amplification was performed in a 20 μL reaction mixture containing 2 μl of corresponding cDNA diluted 1:100, 800 nM of corresponding primers and 2X ddPCR EvaGreen supermix (Bio-Rad). Samples were subjected to droplet generation by an automated droplet generator and end-point PCR was performed afterwards. Cycling steps for the ddPCR were as follows: initial enzyme activation at 95 °C for 10 min followed by 50 cycles of denaturation and annealing (each cycle at 95 °C for 30 s; 58 °C for 1 min; 72 °C for 30 s) and ending with enzyme deactivation at 98 °C for 10 min. Finally, droplets were read on a droplet reader and data were analyzed using QuantaSoft™ Software, which determines the number of droplets that were positive and negative for each fluorophore in each sample. The fraction of positive droplets was then fitted to a Poisson distribution in QuantaSoft™ software to determine the absolute copy number in units of copies/μl. Ratios of *Bsph1* and *Bsph2* were calculated with endogenous control *Hprt*. Primers used are listed in Table 1.

### Fertility assessment

Sexually mature *Bsph1*/*2* DKO male mice (6-8 weeks of age) were paired with two WT female mice for three months. Following pairings, cages were checked every morning for copulatory plugs. Newborn pups were analyzed for numbers and weight at 6 and 21 days, and 6 weeks from birth.

### Epididymal sperm analysis

Epididymides from either WT or *Bsph1/2* DKO mice were dissected, and cauda regions were placed in a petri dish containing 300 μl of human tubal fluid medium (HTF) medium. Multiple incisions were made in the separated cauda to squeeze out sperm, which were then incubated at 37 °C-5% CO_2_ for 10 min. Sperm suspensions (3 μl) were loaded into a Leja slide chamber (20 μm depth) and sperm were analyzed for motility and concentration using Sperm Class Analyzer (SCA) (software version 5.2.0.1.; Barcelona, Spain, MICROPTIC S.L.). The parameters for video utilization were as follows: frames acquired, 50; VCL (rapid), 320 μm/s; VCL (medium), 193 μm/s; VCL (slow), 80 μm/s; VAP set to 7; Lin rapid, 40% and connectivity, 20.

### Liquid chromatography tandem mass spectrometry (LC-MS/MS)

Epididymides from six *Bsph1*/*2* DKO and WT adult male mice (aged 6-7 weeks) were dissected. Total epididymal protein was extracted using TRIzol™ reagent (Invitrogen, Carlsbad, CA, USA), according to the manufacturer’s protocol. Protein concentration was quantified with the Pierce™ BCA Protein Assay Kit (Thermo Fisher Scientific, Bremen, Germany), then 80 μg of each sample was separated by SDS-PAGE on 18% sodium dodecyl sulfate-polyacrylamide gels. Gels were stained with Coomassie Blue and bands corresponding to BSPH1 and BSPH2 (10-20 kDa) were excised and subjected to LC-MS/MS analysis by the Proteomics Core Facility at CHU de l’Université Laval (Québec, Canada), as described previously^14^. The UniProt complete proteome *Mus musculus* Scaffold (version Scaffold_4.8.4, Proteome Software Inc., Portland, OR) was used to interpret MS/MS-based peptide and protein identification.

### Statistical analysis

Statistical calculations were performed using Prism 8 software (GraphPad Software, La Jolla, CA, USA). Statistical analyses were performed using the Student’s t-test and by two-way ANOVA. Differences were considered significant at *P* < 0.05. Normal distribution and homogeneity of variances were tested before running t-test and two-way ANOVA.
